# A highly feasible simulation model based on nipple-preserving pork portions for practicing superficial mass resection

**DOI:** 10.1186/s12893-026-03840-0

**Published:** 2026-05-18

**Authors:** Piaopiao Jin, Xiaoyu Weng, Xiaoyu Zhang, Tian Fang, Lei Geng, Shaohua Chen, Tingbo Liang, Qida Hu

**Affiliations:** 1 Health Management Center, First Affiliated Hospital, Zhejiang University School of Medicine, Hangzhou, 310006 China; 2 Department of Hepatobiliary and Pancreatic Surgery, First Affiliated Hospital, Zhejiang University School of Medicine, Hangzhou, 310006 China; 3 Zhejiang Surgical Residency Training Quality Control Center, Hangzhou, 310006 China; 4 Department of Medical Education, First Affiliated Hospital, Zhejiang University School of Medicine, Hangzhou, 310009 China

**Keywords:** Superficial mass resection, Pork-based simulation model, Surgical training, Competency evaluation, Surgical simulation, Medical education

## Abstract

**Background:**

Superficial mass resection is a core surgical skill requiring practical training. Existing simulation models, such as silicone pads or virtual reality, are often costly or inaccessible, creating a need for an affordable and feasible alternative for surgical education.

**Methods:**

In this feasibility study, we developed a low-cost, high-fidelity simulation model using a rind-on pork belly with preserved nipples to practice this procedure and evaluated its efficacy in a skill training study involving 56 surgery residents, whose competency was assessed using a procedure-specific rating scale and the Direct Observation of Procedural Skills (DOPS) tool.

**Results:**

The pork-based model effectively differentiated competency levels among residents with varying experience. It demonstrated superior fidelity compared to silicone models, without increased operational difficulty, proving particularly suitable for beginners.

**Conclusions:**

This pork-based simulation model is low-cost and easy to prepare, and it represents a high-fidelity training tool, highlighting the possibility of using biological models in hands-on surgical simulation.

**Supplementary Information:**

The online version contains supplementary material available at 10.1186/s12893-026-03840-0.

## Background

Superficial mass resection is a common procedure that surgical residents are required to master. For most surgery residents, the first experience performing superficial mass resection was on a human subject under the supervision of the senior surgeons, following the Halstedian paradigm, other than simulation models, since it is difficult to reproduce similar practice experience with high fidelity for this surgical procedure on a non-human subject. A large-scale educational study has shown that the performance level of surgical residents is time- and skill-dependent [1], underscoring the importance of task repetition in a simulation environment before performing procedures on real patients [2].

Currently, there are a few simulation methods for surgery residents to learn how to perform the superficial mass resection procedure, while addressing the practice of surgical techniques such as incision, tissue dissection, and suturing. The simulated skin pads, particularly the silicone pad, which have been widely available in routine hands-on surgical training, could be used in the simulation of the skin mass, but with low similarity either in appearance or in texture. Advances in printing technologies allow for developing hands-on simulation models for surgical training by 3D printing with various materials [3]. However, 3D-printed simulation models might result in substantial costs due to time-consuming production and heavy demand for specific materials, subsequently increasing the total training cost. Yet there has been no other available hands-on simulation approach, based on modification of simulated skin pads or artificial skin, for the superficial mass resection procedure.

Driven by the virtual reality (VR) technique, surgical simulators allow learners to rehearse complex procedures without risk to patients, providing essential hands-on experience in a safe setting [2]. The VR simulation models for superficial mass resection, created from imaging studies, enable preoperative planning for tutorial purposes [4, 5]. Furthermore, the combination of VR and augmented reality (AR) has emerged as a sophisticated simulation platform, offering interactive 3D models that can be manipulated to simulate various surgical scenarios [6–8]. These technologies have been shown to improve procedural skills and decision-making abilities by offering immediate feedback on technique and outcomes [2]. However, developing VR and AR simulation models requires interactive equipment that runs the virtually constructed 3D models [9]. These additional requirements in hardware, along with extra cost in VR/AR research, have greatly limited the wide use of this simulation approach in the perspectives of economy and feasibility.

Therefore, developing an inexpensive and highly available simulation model for practicing superficial mass resection is strongly required to fulfill the large demand in various surgical teaching circumstances. Herein, we demonstrated an innovative simulation model of rind-on pork belly portions with nipples, demonstrating good feasibility, high cost-effectiveness, and high fidelity for practicing superficial mass resection.

## Methods

### Preparation of the pork-based simulation model

**Fig. 1:**
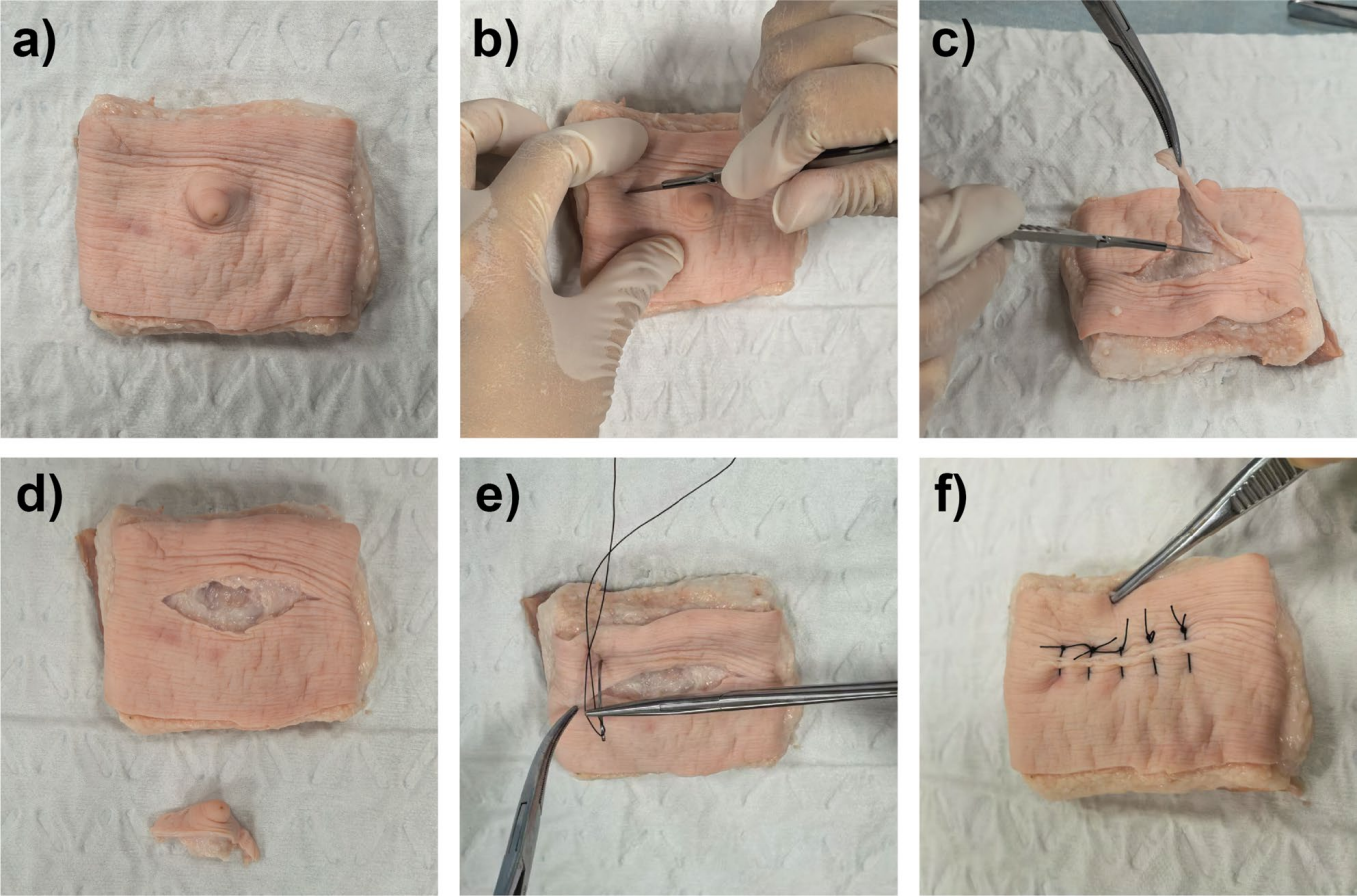
The pork-based simulation model and the simplified procedure steps. **a** The rind-on pork belly portion with a center-located nipple. Preoperative steps were not shown here. **b** Incise the skin tissue. **c** Properly dissect the superficial mass with clamps and scissors. **d** The dissected nipple (“superficial mass”) and the residual simulation model. **e** Suture. **f** Final status followed by wound care

Rind-on pork belly portions were obtained from a commercial porcine meat supplier. The specimens were obtained from the male Large White pigs (*Sus domesticus*) aged at least 1 year, with full hair shaved. Nipples were carefully preserved, and the pork was then cut into square-shaped portions with a side length of 15 cm including a nipple located at the portion center (Fig. 1). Either the nipple alone or the composite structure of the nipple and its corresponding mammary gland, located between the nipples and the underlying lean muscle layer, could serve as the surgical target for superficial mass resection training.

### Feasibility evaluation on model preparation

Our study group purchased the rind-on pork belly portions at 15 Chinese Yuan (about 2.1 US dollars based on the exchange rate as of April 2024) per portion cut. The pork-based model was prepared and stored in a 4 °C refrigerator for no more than 2 days before teaching use, indicating an additional energy consumption cost of 2 kWh (1 Chinese Yuan) per day for model storage. In comparison, our hospital purchased the silicone model from the model manufacturer at the price of 150 Chinese Yuan (about 21 US dollars), but without any additional storage cost. However, the silicone model could be used for 3–5 times of practicing superficial mass resection, which made the cost of single use decrease to 30–50 Chinese Yuan (4.2 to 7.0 US dollars).

Other costs were relatively low. Two to three 2 − 0 silk sutures with needles were used in a complete simulation procedure, costing 5 Chinese Yuan (0.7 US dollar). Scrubbing solutions and sterile materials cost at most 0.25 Chinese Yuan (3 US cents) in a surgical training procedure, and contributed slight to the total expense.

The pork-based model was directly available upon purchasing from the local butcher’s store. Preparation time in the butcher’s store, according to our requirement, was 5–10 s for each portion cut. With the traffic time between the hospital simulation center and the nearby butcher’s store included in time estimation, every training course using the pork-based model needed approximately 30 min of preparation time. In comparison, the silicone model also required the teaching assistants to mark a 0.5 cm black circle before use, which took 5–10 s per training session.

### Practicing superficial mass resection on the simulation model

This feasibility study was designed as a surgical skills training and competency assessment study. The pork-based simulation model can be used for training at varying levels of difficulty. For the beginner trainees, excision of the nipple alone was set as the training goal. As for the advanced trainees, en-bloc radical resection of the nipple, its areola, and the corresponding mammary gland was required to be completed. In detail, a clinical simulation scenario was proposed to the trainees. The trainees would first accomplish preoperative preparations, including evaluation of the superficial mass, determination of the suitable surgical procedure, informed consent, and patient identity check. The trainees would then complete the following steps in order: surgical scrub, skin disinfection, establishing a sterile field, gowning and gloving, local anesthesia, skin incision using the bay leaf technique, mass dissection, specimen removal, layered suturing (simple interrupted sutures for the underlying subcutaneous tissue and Donati’s sutures for the surface tissue were recommended), dressing and bandaging. They were also required to handle postoperative works such as sharps disposal, instrument recycling, and postoperative communications. The trainers would quantitatively rate the trainees’ performance according to the procedure-specific rating scale (Table 1). This comprehensive scale was designed to evaluate performance within a simulated full clinical encounter, encompassing both technical procedural skills (e.g., incision, dissection, suturing) and essential clinical competencies (e.g., communication, preparation). Meanwhile, the competency level of the trainees was also evaluated using Direct Observation of Procedural Skills in Surgery (surgical DOPS, Table 2), which has demonstrated satisfactory validity and reliability in our previous evaluation approach [10].

**Table 1:** Rating scale to trainee’s performance of superficial mass resection

Item	Technical requirements	Scoring details
1. Preoperative preparation (10 points)	1.1. Identity check (3 points)	Oral statement in the simulated setting (3 points).
	1.2. Assessment (7 points)	Using a marker pen to indicate the operation location and the mass margin (7 points).
2. Operation procedure (70 points)	2.1. Planning surgical incision (5 points)	Choose an incision that is parallel to the skin texture or follows the contours of the body surface (5 points).
	2.2. Surgical aseptic technique and sterile field (10 points)	Complete surgical handscrub (1 point), skin disinfection (2 points), gowning (2 points), gloving (2 points), and establishing the sterile field (3 points) in the correct order.
	2.3. Local anesthesia (5 points)	Check the anesthetic agents (1 point), subcutaneously infiltrative administration (3 points), and effectiveness evaluation (1 point)
	2.4. Operation (25 points)	Correctly incise the skin tissue using bay leaf technique (4 points), properly dissect the superficial mass with clamps and scissor (8 points), complete mass resection when distally grasping the mass using Allis forceps (4 points). The other scoring points attribute to skillful performance (3 points), gentle maneuver (3 points), and correct anatomy (3 points). All the 25 points will be deducted if there is residual mass tissue.
	2.5. Layered closure (20 points)	Suture the dermis and underlying subcutaneous tissue (10 points) and surface tissue (10 points) by layer. Five aspects including tensile strength, suture spacing, proper knotting, appositional suture pattern, and absence of dead space, should be evaluated in both layered closures, counted for 2 point in each aspect.
	2.6. Wound care (5 points)	Wound disinfection (2 points) and dressing (3 points).
3. Postoperative care (10 points)	3.1. Surgical instruments (2 points)	Recycle surgical instruments (1 point) and discard sharps in a sharps box (1 point).
	3.2. Postoperative conversations (8 points)	Complete informed consent of operation summary (4 points) and aftercare details (4 points) to the patient.
4. Asepsis (10 points)	Follow the aseptic principles (10 points).	

**Table 2:** Surgical DOPS for performance evaluation of superficial mass resection

Difficulty of the procedure: Average difficulty		
Assessor’s comments on this activity	Rating†	Specific comments
− 1. Describes indications, relevant anatomy, & details of procedure		
− 2. Obtains informed consent, after explaining procedure & comps		
− 3. Prepares for procedure, checks for instruments		
− 4. Gets patient history, administers effective analgesia or safe sedation		
− 5. Proper draping and demonstrates good asepsis		
− 6. Handles tissue gently		
− 7. Enters correct plane, hemostasis		
− 8. Closure of space, appropriate suturing		
− 9. Techniques up to level of training and safe use of instruments		
− 10. Deals with any unexpected event or seeks help when appropriate		
− 11. Completes required documentation (written or dictated)		
− 12. Issues clear post-procedure instructions to patient and/or staff		
Feedback (general, strengths, improvement needs, recommended actions)		
Global summary (performance levels)		
- Level 0. Insufficient evidence observed to support a summary judgement		
- Level 1. Unable to perform the procedure, or part observed, under supervision		
- Level 2. Able to perform the procedure, or part observed, under supervision		
- Level 3. Able to perform the procedure with minimum supervision (needed occasional help)		
- Level 4. Competent to perform the procedure unsupervised (could deal with complications that arose)		

*N* Not observed, *I* Improvement required, *S* Satisfactory, *O* Outstanding

† Ratings

As for the evaluation of the competency performance in superficial mass resection, 56 surgery residents from postgraduate year (PGY)-one to PGY-3 were divided into work groups consisting of a trainee surgeon and an assistant. The trainee surgeon and the assistant in the work group were at the same postgraduate year. After the trainee surgeon completed practice, the two residents swapped roles, to ensure every trainee resident has equal opportunities to practice superficial mass resection.

### Model comparison

The pork-based simulation model was compared with the regular simulation model. The regular model was established on a silicone pad with a 0.5 cm black circle mark at the pad’s center to indicate the superficial mass. All fifty-six residents using the pork-based models were required to complete superficial mass resection using the silicone models. After the simulated training, feedback questionnaires (Supplementary Table S1) were retrieved from all the trainees who were required to rate three scales, namely fidelity, operational difficulty, and overall satisfaction, on a scale of 1 to 10. The ratings for these three scales were then compared across the two models. The model comparison was perception-based only, because no objective performance data were collected for the silicone model.

### Statistical analysis

Data are presented as mean ± standard deviation (SD). Statistical calculations were made using Prism 7 (GraphPad, La Jolla, CA) with the indicated analytical methods. *p* < 0.05 was considered significant.

## Results

### Comparison of competency performance using the pork-based simulation model

**Fig. 2:**
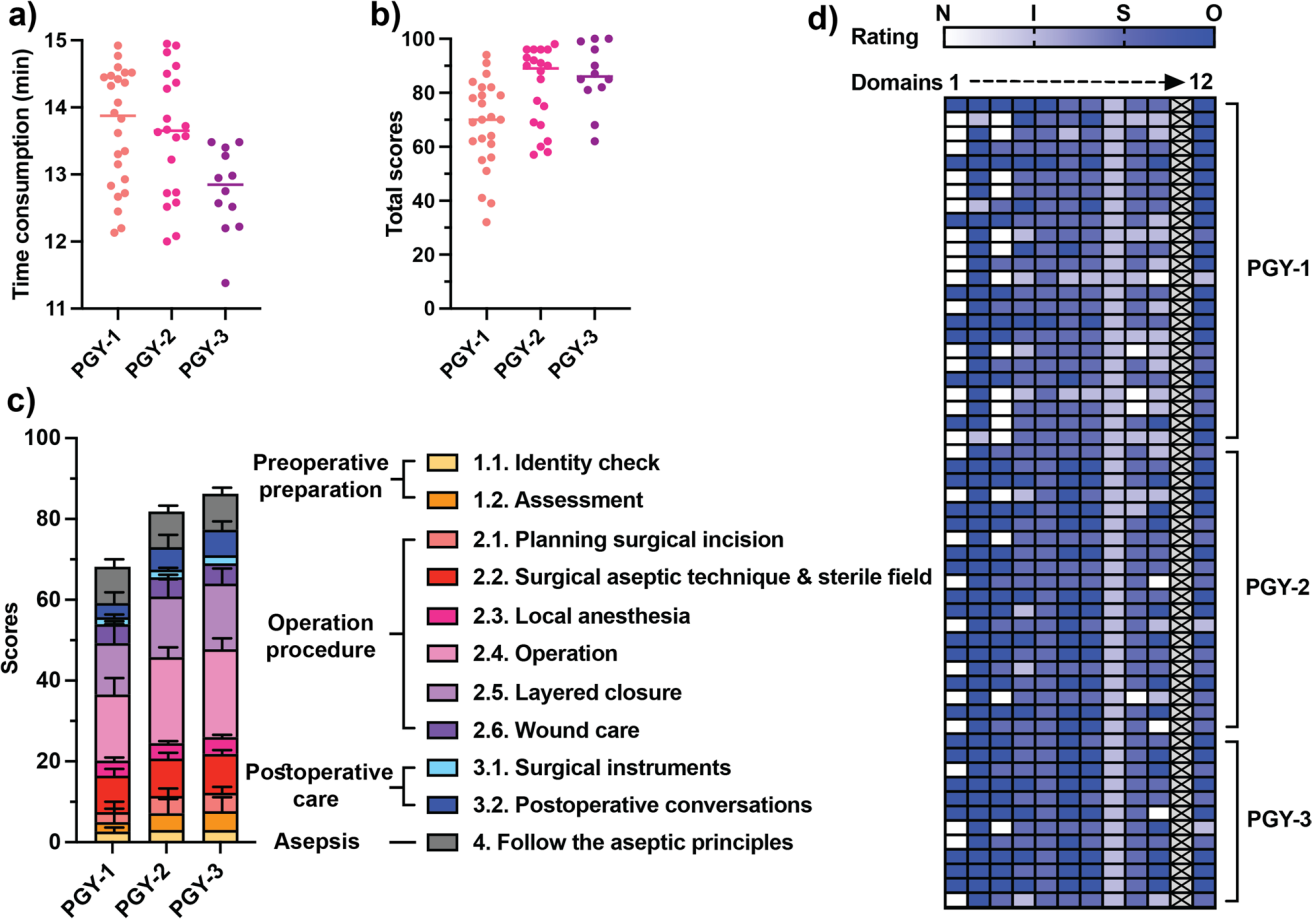
The pork-based simulation model enables stratification among different PGY groups. Comparison analyses of **a** completion time, **b** distribution of the total scores, and **c** score compositions by procedure steps in different PGY groups were shown. **d** Domain-specific heatmap presentation for different PGY residents evaluated by surgical DOPS)

A total of 56 trainees, consisting of 24 PGY-1 (14 males and 10 females), 20 PGY-2 (14 males and 6 females), and 12 PGY-3 (8 males and 4 females) surgery residents, were tested for their competency performance of superficial mass resection on the pork-based simulation model, according to the procedure-specific rating scale and surgical DOPS (Table 3). All the trainees were able to complete the simulated operation on the pork-based model in 15 min, but the mean operation time of the PGY-3 residents was shorter than the other residents (12.77 ± 0.63 min for PGY-3; vs. 13.61 ± 0.94 min for PGY-2, *p* = 0.02; vs. 13.69 ± 0.89 min for PGY-1, *p* = 0.01; Tukey’s test post one-way ANOVA; Fig. 2a). Their final scores varied from 32 to 100, and the performance scores of the PGY-2 and PGY-3 residents were significantly higher than those of PGY-1 residents (68.17 ± 12.14 points for PGY-1; vs. 81.85 ± 14.58 points for PGY-2, *p* = 0.01; vs. 86.25 ± 12.14 points for PGY-3, *p* < 0.01; Tukey’s test), suggesting the pork-based simulation model be able to stratify different levels of surgical competency performance (Fig. 2b; Table 3). Interestingly, the PGY-3 residents achieved slightly, but not significantly, higher performance scores compared to the PGY-2 residents (*p* = 0.70; Tukey’s test), indicating that superficial mass resection is suitable for basic surgical training rather than an advanced training program established for the residents in PGY-2 and PGY-3. Among all the sub-scoring items, surgical technique-related contents, including skin incision, mass dissection, and specimen removal, yielded greater discernibility than other procedure-related contents such as aseptic preparation and dressing (Fig. 2c; Table 3). Meanwhile, the evaluation outcomes between different PGY residents using the surgical DOPS tool (Fig. 2d) confirmed that PGY-2 and PGY-3 residents had better competency performance quantified in global summary level in comparison to PGY-1 residents (2.13 ± 1.19 for PGY-1; vs. 2.95 ± 1.15 for PGY-2, *p* = 0.04; vs. 3.42 ± 0.79 for PGY-3, *p* < 0.01; Tukey’s test).

**Table 3:** Detailed scores in each procedure step according to the procedure-specific rating scale

Sub-score items	PGY-1 *n* = 24	PGY-2 *n* = 20	PGY-3 *n* = 12
1.1. Identity check (3 points)	2.6 ± 1.0	3.0 ± 0.0	3.0 ± 0.0
1.2. Assessment (7 points)	2.3 ± 3.4	4.2 ± 3.5	4.7 ± 3.4†
2.1. Planning surgical incision (5 points)	2.5 ± 2.6	4.3 ± 1.8	4.6 ± 1.4†
2.2. Surgical aseptic technique & sterile field (10 points)	9.0 ± 1.7	9.3 ± 1.4	9.6 ± 1.0
2.3. Local anesthesia (5 points)	3.8 ± 0.7	3.8 ± 0.5	4.2 ± 0.6
2.4. Operation (25 points)	16.4 ± 4.0	21.3 ± 2.4†	21.8 ± 2.7†
2.5. Layered closure (20 points)	12.7 ± 4.7	15.0 ± 4.3	16.3 ± 3.8
2.6. Wound care (5 points)	4.7 ± 0.8	4.8 ± 0.6	5.0 ± 0.0
3.1. Surgical instruments (2 points)	1.7 ± 0.7	1.9 ± 0.5	2.0 ± 0.0
3.2. Postoperative conversations (8 points)	3.5 ± 2.7	5.6 ± 3.0	6.3 ± 2.1
4. Follow the aseptic principles (10 points).	9.0 ± 1.9	8.8 ± 1.5	8.9 ± 1.5
Total scores	68.2 ± 16.5	81.9 ± 14.6†	86.3 ± 12.1†

† *p* < 0.05, vs. PGY-1; one-way ANOVA followed by Tukey’s test

### Model comparison in fidelity and operational difficulty

To compare the pork-based model with the silicone model in terms of fidelity, we analyzed the subjective ratings from the retrieved feedback questionnaires. Comparison analyses revealed that the pork-based model resulted in significantly greater fidelity than the silicone model (9.7 ± 0.5 for the pork-based model vs. 5.1 ± 1.9 for the silicone model, *p* < 0.01; paired t test), indicating that this ultra-fieldable simulation, with a nearly full-score rating was well recognized as an acceptable replacement for human tissue. However, the fidelity improvement did not lead to additional operational difficulty (7.0 ± 1.4 for the pork-based model vs. 7.0 ± 1.6 for the silicone model, *p* = 0.99; paired t test). Consequently, the overall satisfaction rating from the residents showed that the pork-based model outperformed the silicone model (9.9 ± 0.3 for pork-based model vs. 4.5 ± 1.3 for silicone model, *p* < 0.01; paired t test). Since no objective performance data were collected for the silicone model, current model comparison with perception-based data might need further validation using performance-related objective ratings.

## Discussion

This study presents a novel, pork-based simulation model for superficial mass resection, highlighting its high feasibility, excellent cost-effectiveness, and enhanced fidelity. The pork-based model provides a low-cost, accessible, and realistic alternative to conventional training tools, including physical simulators, such as silicone models, and technologically advanced VR/AR systems.

Fidelity in simulation refers to how well training replicates real clinical tasks [11]. In this study, trainee feedback yielded near-maximum fidelity ratings for the pork-based model, which significantly surpassed those for the silicone model. This highlights the model’s compelling anatomical and tactile realism. The preserved nipple and mammary gland structures embedded in the pork tissue mirror the physical properties encountered during real superficial mass resections. Unlike silicone pads, which may inadequately mimic tissue planes, resistance, or vascularity [12], the pork model provides variable tissue density and layering that closely simulate the dissection challenges in human skin and subcutaneous tissue. This feature likely explains why the surgical technique subcomponents showed greater variability across PGY levels, indicating that our model is sensitive to skill levels and thus suitable for competency stratification. The findings resonate with previous literature which affirms that animal tissue models, such as porcine skin, offer superior tactile feedback compared with synthetic models [13–15]. The realism of haptic feedback is critical in enhancing psychomotor skill acquisition, especially in the early stages of surgical training [16], as it allows trainees to perceive tissue tension and control dissection depth, bridging the gap between simulation and real operations [17]. Furthermore, the pork-based models are also available for interaction with energy devices, such as an ultrasound scalpel. Additionally, the higher fidelity did not increase perceived operational difficulty, indicating the model provides a realistic yet approachable environment for skill development without overwhelming beginners, countering concerns that high fidelity may obscure fundamental principles.

Balancing high-fidelity training with affordability and widespread availability is a challenging problem in surgical simulation. Virtual reality simulators and sophisticated synthetic models, although pedagogically promising, often come with substantial hardware requirements and ongoing maintenance or licensing costs [18–20]. Our pork-based model, in contrast, is built on locally accessible and inexpensive materials, enabling its wide deployment, including in underfunded or resource-limited teaching environments. The feasibility advantage also lies in its ease of procurement and preparation. Unlike VR/AR simulators that demand expensive headsets, haptic devices, and custom-designed software, the pork-based model is devoid of technological prerequisites and does not require internet connectivity, power-intensive workstations, or technical support. The enhanced portability and adaptability across diverse clinical settings would satisfy the need for cost-effective simulation models in surgical education [21, 22], especially in low- and middle-income countries [23].

Although this study introduces an innovative approach to surgical simulation by integrating biological realism with structured procedural training, there are still some limitations that need to be acknowledged. Firstly, this study was conducted in a single institution with a relatively small subgroup sample size, which constrains its generalization to other settings or learner populations. Secondly, the assessment was short-term, with no follow-up on skill retention, which may make it difficult to assess its impact on the operator’s long-term surgical skills, especially whether training using this model improves performance in real clinical procedures. Third, the current study primarily demonstrated the feasibility and face and construct validity of the pork-based model, leaving several important aspects of simulation model validation unaddressed. In particular, high fidelity and satisfaction received from the trainees are subjective and may be influenced by factors such as the perceived realism of biological tissue, trainee expectations, and the absence of blinding in the evaluation process. Meanwhile, the parallel performance data evaluating the trainees using the silicone model was absent, and this methodological asymmetry led to a lack of a controlled training comparison between the pork-based and silicone models, which may also affect the accuracy of the results, which were primarily limited to subjective ratings such as fidelity, difficulty, and trainee satisfaction. Finally, this pork-based simulation model, while offering high anatomical and haptic fidelity for practicing core surgical maneuvers, cannot fully replicate certain pathological variations found in clinical practice, such as infected or malignant superficial masses, which positioned the model as a fundamental trainer for simulating the generalizable technical challenge of identifying, isolating, and completely excising a subcutaneous target while preserving surrounding tissue integrity. Thus, further multicenter, longitudinal studies with comprehensive comparative data assessing learning progression and skill transfer to clinical practice would be valuable for confirming the educational impact of this simulation model.

## Conclusions

In this feasibility study, the rind-on pork belly simulation model presented in this study offers a feasible, cost-effective, and high-fidelity training tool for superficial mass resection. It demonstrated higher fidelity and trainee satisfaction than the standard silicone model in this single-institution study and may thus serve as a practical, low-cost alternative for surgical skills training among surgical residents.

## Supplementary Information

Supplementary Material 1

## Data Availability

The data that support the findings of this study are available upon reasonable request to Dr. Qida Hu (Email: huqida@zju.edu.cn).

## Abbreviations

VR: Virtual Reality
DOPS: Direct Observation of Procedural Skills
AR: Augmented Reality
PGY: Postgraduate Year
SD: Standard Deviation

## References

[1] Shebrain S, Coster S, Alfred A, De Cecco D, Khalil S, Munene G, Elian A, Timmons J, Sawyer RG. Resident autonomy and performance independence in surgical training are time- and skill-dependent. J Surg Res. 2022;279:285–95. 35802943 10.1016/j.jss.2022.06.027

[2] Seymour NE, Gallagher AG, Roman SA, O’Brien MK, Bansal VK, Andersen DK, Satava RM. Virtual reality training improves operating room performance: Results of a randomized, double-blinded study. Ann Surg. 2002;236(4):458–63. discussion 463 – 454. 12368674 10.1097/00000658-200210000-00008 PMC1422600

[3] Przadka M, Pajak W, Kleinrok J, Pec J, Michno K, Karpinski R, Baj J. Advances in 3D Printing Applications for Personalized Orthopedic Surgery: From Anatomical Modeling to Patient-Specific Implants. J Clin Med. 2025;14(11). 10.3390/jcm14113989 PMC12156138 40507750

[4] Peng MJ, Chen HY, Chen P, Tan Z, Hu Y, To MK, He E. Virtual reality-based surgical planning simulator for tumorous resection in FreeForm Modeling: an illustrative case of clinical teaching. Quant Imaging Med Surg. 2024;14(2):2060–8. 38415160 10.21037/qims-23-1151 PMC10895132

[5] Edwards TC, Soussi D, Gupta S, Khan S, Patel A, Patil A, Liddle AD, Cobb JP, Logishetty K. Collaborative team training in virtual reality is superior to individual learning for performing complex open surgery: A randomized controlled trial. Ann Surg. 2023;278(6):850–7. 37638414 10.1097/SLA.0000000000006079 PMC10631503

[6] McKnight RR, Pean CA, Buck JS, Hwang JS, Hsu JR, Pierrie SN. Virtual reality and augmented reality-translating surgical training into surgical technique. Curr Rev Musculoskelet Med. 2020;13(6):663–74. 32779019 10.1007/s12178-020-09667-3 PMC7661680

[7] Abdel Al S, Chaar MKA, Mustafa A, Al-Hussaini M, Barakat F, Asha W. Innovative surgical planning in resecting soft tissue sarcoma of the foot using augmented reality with a smartphone. J Foot Ankle Surg. 2020;59(5):1092–7. 32505724 10.1053/j.jfas.2020.03.011

[8] Dhillon J, Tanguilig G, Kraeutler MJ. Virtual and augmented reality simulators show intraoperative, surgical training, and athletic training applications: A scoping review. Arthroscopy. 2025;41(2):505–15. 38387769 10.1016/j.arthro.2024.02.011

[9] Venkatesan M, Mohan H, Ryan JR, Schurch CM, Nolan GP, Frakes DH, Coskun AF. Virtual and augmented reality for biomedical applications. Cell Rep Med. 2021;2(7):100348. 34337564 10.1016/j.xcrm.2021.100348 PMC8324499

[10] Jia J, Yu J, Geng L, Bai X, Liang T, Zheng S. The application of DOPS in the surgical skill training of general surgery specialists (in Chinese). Adv Educ. 2020;10(3):279–83.

[11] Birbara NS, Pather N. Real or not real: The impact of the physical fidelity of virtual learning resources on learning anatomy. Anat Sci Educ. 2021;14(6):774–87. 33002293 10.1002/ase.2022

[12] Silva A, Rodriguez JER, Oliveira MC, Negreiros RMA, Cavalcante LP. The alternative model of silicone for experimental simulation of suture of living tissue in the teaching of surgical technique. Acta Cir Bras. 2019;34(4):e201900410. 31038587 10.1590/s0102-865020190040000010 PMC6583932

[13] DeMasi SC, Katsuta E, Takabe K. Live animals for preclinical medical student surgical training. Edorium J Surg. 2016;3(2):24–31. 28713875 PMC5509225

[14] Acea Nebril B, Garcia Novoa A, Bouzon Alejandro A, Centeno Cortes A. Porcine model for training in oncoplastic breast surgery technical description and results of its application in a training course in oncoplastic and reconstructive techniques in breast surgery. J Plast Reconstr Aesthet Surg. 2019;72(6):1030–48. 30658949 10.1016/j.bjps.2018.12.049

[15] Lee JS, Lee J, Kim YH, Park HY, Yang JD. Porcine training models for endoscopic and robotic reconstructive breast surgery: a preliminary study. Gland Surg. 2021;10(8):2346–53. 34527546 10.21037/gs-21-398 PMC8411080

[16] Strom P, Hedman L, Sarna L, Kjellin A, Wredmark T, Fellander-Tsai L. Early exposure to haptic feedback enhances performance in surgical simulator training: a prospective randomized crossover study in surgical residents. Surg Endosc. 2006;20(9):1383–8. 16823652 10.1007/s00464-005-0545-3

[17] Jordan GH. Techniques of tissue handling and transfer. J Urol. 1999;162(3 Pt 2):1213–7. 10458469 10.1016/S0022-5347(01)68137-0

[18] Khor WS, Baker B, Amin K, Chan A, Patel K, Wong J. Augmented and virtual reality in surgery-the digital surgical environment: Applications, limitations and legal pitfalls. Ann Transl Med. 2016;4(23):454. 28090510 10.21037/atm.2016.12.23 PMC5220044

[19] Mao RQ, Lan L, Kay J, Lohre R, Ayeni OR, Goel DP, Sa D. Immersive virtual reality for surgical training: A systematic review. J Surg Res. 2021;268:40–58. 34284320 10.1016/j.jss.2021.06.045

[20] Rogers MP, DeSantis AJ, Janjua H, Barry TM, Kuo PC. The future surgical training paradigm: Virtual reality and machine learning in surgical education. Surgery. 2021;169(5):1250–2. 33280858 10.1016/j.surg.2020.09.040

[21] Ziv A, Wolpe PR, Small SD, Glick S. Simulation-based medical education: An ethical imperative. Simul Healthc. 2006;1(4):252–6. 19088599 10.1097/01.SIH.0000242724.08501.63

[22] Cook DA, Andersen DK, Combes JR, Feldman DL, Sachdeva AK. The value proposition of simulation-based education. Surgery. 2018;163(4):944–9. 29452702 10.1016/j.surg.2017.11.008

[23] Abahuje E, Tuyishime E, Alayande BT. Global surgical simulation education, current practices, and future directions. Surgery. 2025;180:109050. 39740603 10.1016/j.surg.2024.109050

