# Supplementary material for: A highly feasible simulation model based on nipple-preserving pork portions for practicing superficial mass resection
Source: BMC Surg. 2026 May 18;26:461. doi: 10.1186/s12893-026-03840-0 (PMC13348459; doi:10.1186/s12893-026-03840-0)
Supplement: Supplementary file 1 — Supplementary Material 1 [file 12893_2026_3840_MOESM1_ESM.docx]

**Supplementary Materials**

**Table S1. Questionnaire on Superficial Mass Excision Simulation Training for Residents**

**Section 1: Basic Information**

*(Please check √ or fill in the blanks as applicable)*

1. Name/ID (optional): _________________________
2. Training year:

□ Postgraduate Year 1 (PGY-1)

□ Postgraduate Year 2 (PGY-2)

□ Postgraduate Year 3 (PGY-3) or above

1. Prior experience with similar simulation training:

□ None

□ 1–2 times

□ 3–5 times

□ >5 times

**Section 2: Experience with the Pork-Based Simulation Model**

*(Recall your experience using the* ***pork-based model*** *for superficial mass excision training. Rate the following dimensions on a scale of 1–10, where 1 = Very Poor/Very Difficult/Very Dissatisfied and 10 = Excellent/Very Easy/Very Satisfied.)*

| **Evaluation Dimension** | **Score (1–10)** |
| --- | --- |
| Fidelity | □1 □2 □3 □4 □5 □6 □7 □8 □9 □10 |
| Operational Difficulty | □1 □2 □3 □4 □5 □6 □7 □8 □9 □10 |
| Overall Satisfaction | □1 □2 □3 □4 □5 □6 □7 □8 □9 □10 |

**Section 3: Experience with the Silicone Simulation Model**

*(Recall your experience using the* ***regular silicone model*** *for superficial mass excision training. Rate the following dimensions on a scale of 1–10, where 1 = Very Poor/Very Difficult/Very Dissatisfied and 10 = Excellent/Very Easy/Very Satisfied.)*

| **Evaluation Dimension** | **Score (1–10)** |
| --- | --- |
| Fidelity | □1 □2 □3 □4 □5 □6 □7 □8 □9 □10 |
| Operational  Difficulty | □1 □2 □3 □4 □5 □6 □7 □8 □9 □10 |
| Overall Satisfaction | □1 □2 □3 □4 □5 □6 □7 □8 □9 □10 |

**Thank you for your participation! Your feedback is critical for refining our simulation training programs.**
